# Mate selection and current trends in the prevalence of autism

**DOI:** 10.1186/s13229-024-00607-3

**Published:** 2024-07-16

**Authors:** Elizabeth Forsen, Natasha Marrus, Jacqueline Joyce, Yi Zhang, John N. Constantino

**Affiliations:** 1 Washington University in St. Louis, St. Louis, MO USA; 2 Department of Psychiatry, Washington University in St. Louis, St. Louis, MO USA; 3 Emory University, Atalanta, GA 30322 USA; 4 Department of Behavioral and Mental Health, Children’s Healthcare of Atlanta, Atalanta, GA 30322 USA

**Keywords:** Autism, Autism prevalence, Assortative mating, Mate selection, Racial and ethnic minorities

## Abstract

**Background:**

According to the most recent U.S. CDC surveillance data, the rise in prevalence of childhood autism spectrum disorder among minority children has begun to outpace that of non-Hispanic white children. Since prior research has identified possible differences in the extent of mate selection for autistic traits across families of different ethnicity, this study examined variation in autism related traits in contemporaneous, epidemiologically ascertained samples of spousal pairs representing Hispanic and non-Hispanic white populations. The purpose was to determine whether discrepancies by ethnicity could contribute to differential increases in prevalence in the current generation of young children.

**Methods:**

Birth records were used to identify all twin pairs born between 2011 and 2013 in California and Missouri. Families were selected at random from pools of English-speaking Hispanic families in California and Non-Hispanic White families in Missouri. Autistic trait data of parents was obtained using the Adult Report Form of the Social Responsiveness Scale (SRS-2).

**Results:**

We did not identify a statistically significant difference in the degree of mate selection for autism related traits between Hispanic and non-Hispanic white spousal pairs. However, the degree of spousal correlation observed in this recent cohort was pronounced (on the order of ICC 0.45) and exceeded that typically reported in prior research (on the order of 0.30), surpassing also widely reported estimates for sibling correlation (also on the order of 0.30).

**Limitations:**

The sample did not allow for a direct appraisal of change in the magnitude of spousal correlation over time and the ascertainments of trait burden were derived from spouse report.

**Conclusion:**

Across two epidemiologically ascertained samples of spousal pairs representing Hispanic and non-Hispanic white families across two U.S. states (respectively, California and Missouri), the extent of autism-related trait co-variation for parents of the current generation of young children is substantial and exceeds correlations typically observed for siblings. Given the heritability of these traits and their relation to autism risk, societal trends in the degree of mate selection for these traits should be considered as possible contributors to subtle increases in the incidence of autism over time and across generations.

**Supplementary Information:**

The online version contains supplementary material available at 10.1186/s13229-024-00607-3.

## Introduction

A recent report from the U.S. Center for Disease Control’s (CDC) Autism and Developmental Disabilities Monitoring (ADDM) Network revealed that the prevalence of childhood autism spectrum disorder (ASD) is continuing to increase across all population groups, with the most recent data (2012 birth cohort) estimating a prevalence of 1 in 36 among 8-year-olds in the U.S. [1]. An important trend in the surveillance data over the past decade [2] is that the prevalence of ASD among minority children has not only “caught up” with that of non-Hispanic whites (NHW), but is now starting to outpace NHW prevalence. Among 8-year-olds in the 2012 birth cohort, the prevalence of ASD was 31.6, 24.3, and 29.3 for Hispanic, White-Non-Hispanic, and Black-Non-Hispanic populations, respectively [1]. This trend was particularly pronounced in Maryland, where a Black child was 2.0 × as likely as a white child to be diagnosed with ASD [1]. See Supplemental Table 2 for prevalence by race and ethnicity across the ADDM birth cohort.

There are a number of factors that can increase diagnostic prevalence of a predominantly-inherited condition over time and across generations. One that may differentially impact populations across disparate ancestral origins or cultures is the phenomenon of assortative (preferential) mating. Assortative mating has been repeatedly documented on the basis of greater-than-expected spousal correlations for highly-heritable autistic traits in the general population [3–5], and among the parents of children affected by autism [4,^5^]. Excess sharing of polygenic risk specific to autism has been confirmed among spousal pairs in families affected by autism in prior molecular genetic analyses [3, 6], and the degree of phenotypic correlation for autistic traits among parents of children with autism was observed to be particularly pronounced among Hispanic spousal pairs (ICC = 0.60) in a Miami, FL study [7]. Across generations, mate selection for likeness in a given trait increases diversity-of-outcomes of offspring in the next generation and in particular can increase the prevalence of offspring expressing phenotypic traits at the higher and lower extremes of the distribution for their generation.

In this analysis, we leverage a contemporary and simultaneously-ascertained epidemiologic cohort of parents in Missouri and California to provide updated information on the degree to which spousal pairs exhibit correlations in autism-related trait variation in Hispanic (California) and non-Hispanic (Missouri) US populations, to provide a contemporary context for the new information reported by ADDM, and to propose consideration of the tracking of this phenomenon as a potential influence on ASD prevalence in the US.

## Methods

### Sample

Quantitative autistic trait data of parents of epidemiologically ascertained toddler twins were collected in the Early Quantitative Characterization of Reciprocal Social Behavior Study (HD068479) in Missouri and California. Birth records were used to identify all twin pairs born between 2011 and 2013 in those respective States, and parents were selected at random from pools of self-identified English-speaking Hispanic families in California and Non-Hispanic White families in Missouri to be contacted for enrollment in the study. Details of subject ascertainment are described in prior publications of the results of this study—there were no documented differences in demographic characteristics of parents across the two states except for higher levels of maternal education in the Missouri cohort [8, 9]. Demographic Information for Study Samples and State Census Data is provided in Supplemental Table 3.

### Measures

Quantitative autistic trait data was obtained using the Adult Report Form of the Social Responsiveness Scale, second edition (SRS-2) [10]. In Missouri, 95 spousal pairs completed the cross-informant-report SRS-2 (mothers reporting on fathers and fathers reporting on mothers) and in California, 93 spousal pairs completed the SRS-2. Higher scores on the SRS-2 reflect a greater extent to which parents carry sub-clinical traits which at the phenotypic extreme characterize autism spectrum disorders.

### Data analysis

Intraclass correlation coefficients (ICCs) for square-root-transformed SRS-2 scores between spouses were calculated to index assortative mating. Quantile regressions tested the relationship between spousal SRS-2 scores based on estimates of regression coefficients at the 25th, 50th, and 75th quantile.

## Results

Mean SRS score did not significantly differ between California and Missouri mothers (CA = 28.7 (17.1), MO = 25.7 (18.5), *p* = 0.25), while California fathers showed significantly increased mean SRS score compared to Missouri fathers (CA = 26.6 (18.4), MO = 19.6 (15.6), *p* = 0.005). Histograms depicting the distribution of SRS total and subscale scores for mothers and fathers are presented in Supplemental Fig. 1. As shown in Supplemental Fig. 3 and Supplemental Table 4, there was no evidence of score discrepancies between spouses differing between sites or predicting child SRS scores.

**Fig. 1:**
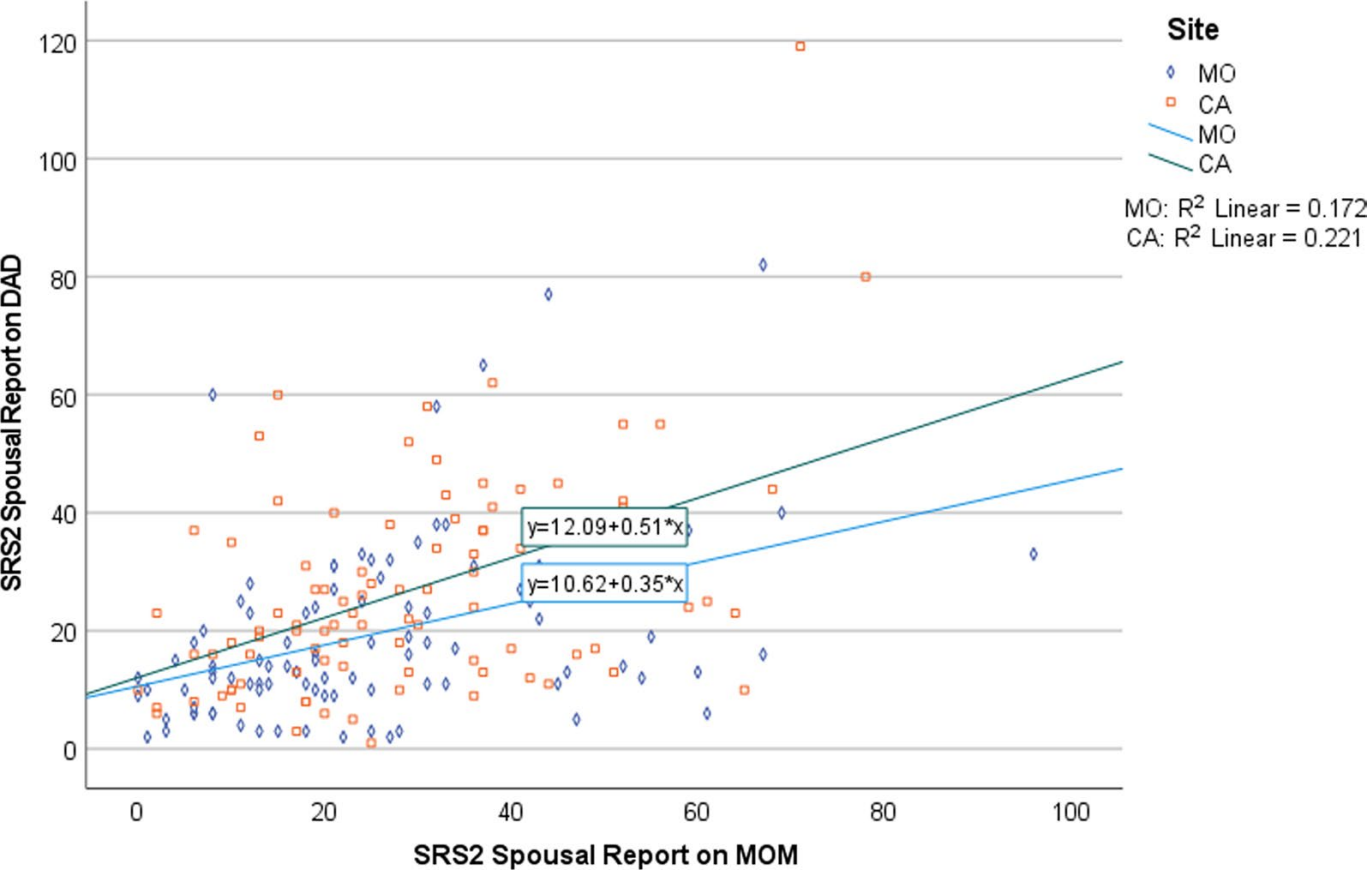
Spousal SRS-2 pearson’s correlations among Hispanic (California) and non-hispanic (Missouri) parents

Missouri spousal pairs exhibited an intra class correlation (ICC) of 0.43 (95% CI: 0.25, 0.58); the ICC for California spousal pairs was 0.44 (95% CI: 0.26, 0.59); a scatter plot of the paired data is presented in Fig. 1. A summary of the respective intraclass correlations by site for the SRS subscales is presented in Supplemental Table 1.

Quantile regression analysis showed that stronger associations between spousal SRS scores were.

successively observed at higher quantiles (i.e., level of autistic traits). Significant spousal associations were observed at each quantile, with increases in beta coefficients from the 25th through the 75th quantile for both the MO (b_25_ = 0.15, b_50_ = 0.30, b_75_ = 0.42, p’s < 0.03) and CA sites (b_25_ = 0.24, b_50_ = 0.54, b_75_ = 0.78, p’s < 0.02). A plot depicting the respective regression lines for each quantile is provided in Supplemental Fig. 2.

## Discussion

Although we did not observe a statistically-significant difference between the Hispanic California sample and the non-Hispanic White Missouri sample, these data provide strong evidence of the effects of variation in quantitative autistic traits on mate selection in two contemporary epidemiologically-ascertained populations. Persistent effects of preferential mating for the characterizing traits and features of autism, across time and generations, can contribute to the clinical-level aggregation of autistic traits among offspring; for example, a previous epidemiologic study demonstrated that pairings of parents in the upper quintile for autistic traits was associated with a doubling of the rate for clinical-level ASD among offspring [5]. The current data reflect a significantly higher degree of spousal correlation than has been repeatedly observed in epidemiologic studies of the prior generation (3, 5; ICCs on the order of 0.30–0.35)—with correlations progressively stronger at higher quantiles for autistic trait burden of parents in this sample—but not as high as was observed among parents of Hispanic children with autism in a Miami sample [7]. This analysis captures variation in spousal pairs *within the last decade*, effectively describing that variation in social responsiveness observed in the prior generation (married in the mid-1990s) is still present in the population and possibly intensifying. Given the highly heritable nature of ASD and autistic traits, our analysis suggests assortative mating could conceivably play a role in the observed increase of ASD prevalence among recent birth cohorts from CDC surveillance data. When considering the various contributing factors of changing ASD prevalence rates at the general U.S. and subpopulation-level, investigating spousal correlations may capture important underlying features of the mechanisms driving change in the prevalence of ASD.

While steady increases in diagnostic prevalence for ASD documented by the CDC over the past two decades substantially reflect improved community identification, relative increases in prevalence rates among minority subpopulations, as recently observed, constitute an important public health concern. Factors that serve to increase prevalence in minority populations are highly relevant to existing health disparities. For example, non-white children with autism bear a disproportionate burden of intellectual disability, which is believed to relate to disparities in access to appropriate developmental services and are more likely to depend on public resources for intervention [11–13]. Thus, the findings of the CDC surveillance report coupled with the results of this analysis highlight the pressing need to understand the complex mechanisms underlying the prevalence and severity of autism and its comorbidities.

## Conclusion

With this communication, we capitalize on a contemporary epidemiologic cohort of parents in California and Missouri to investigate potential drivers of a surprising trend in prevalence of autism among minority children. We confirmed evidence for a substantial degree of preferential mating in contemporary parent samples, in both an Hispanic and Non-Hispanic cohort. Increased prevalence in minority populations is of particular concern due to significant racial inequalities in access to quality services for children with ASD. An immediate public health priority is to further explore factors impacting autism prevalence in order to identify appropriate strategies for mitigating the burden of these disparities on minority populations and improving developmental outcomes for children with high familial liability for autism.

### Supplementary Information

Supplementary file 1: SRS, SCI, and RRB T-Scores Among Hispanic (California) and Non-Hispanic (Missouri) Parents.

Supplementary file 2: Respective spousal regression lines when dividing the sample into thirds, by autistic trait burden. California (left), Missouri (right).

Supplementary file 3: Distributions of Parent Score Differences.

Supplementary file 4: Spousal SRS Subscale Intraclass Correlations Among Hispanic (California) and Non-Hispanic (Missouri) Parents.

Supplementary file 5: Autism prevalence at age 8-years by Race and Ethnicity among successive birth cohorts in the Autism and Developmental Disabilities Monitoring (ADDM) program of the U.S. Center for Disease Control.

Supplementary file 6: Demographic Information for Study Samples and State Census Data.

Supplementary file 7: Correlations of Parent Scores with Child Scores in Missouri, California, and both sites, respectively (36 or 48 months).

## Data Availability

All data generated or analyzed during this study are included in this published article and references list.

## Abbreviations

ICC: Intraclass correlation
ADDM: Autism and developmental disabilities monitoring
SRS-2: Social responsiveness scale, second edition
