# Supplementary material for: Mate selection and current trends in the prevalence of autism
Source: Mol Autism. 2024 Jul 16;15:29. doi: 10.1186/s13229-024-00607-3 (PMC11251233; doi:10.1186/s13229-024-00607-3)
Supplement: Supplementary file 1 — SRS, SCI, and RRB T-Scores Among Hispanic (California) and Non-Hispanic (Missouri) Parents. [file 13229_2024_607_MOESM1_ESM.pptx]

## Slide 1
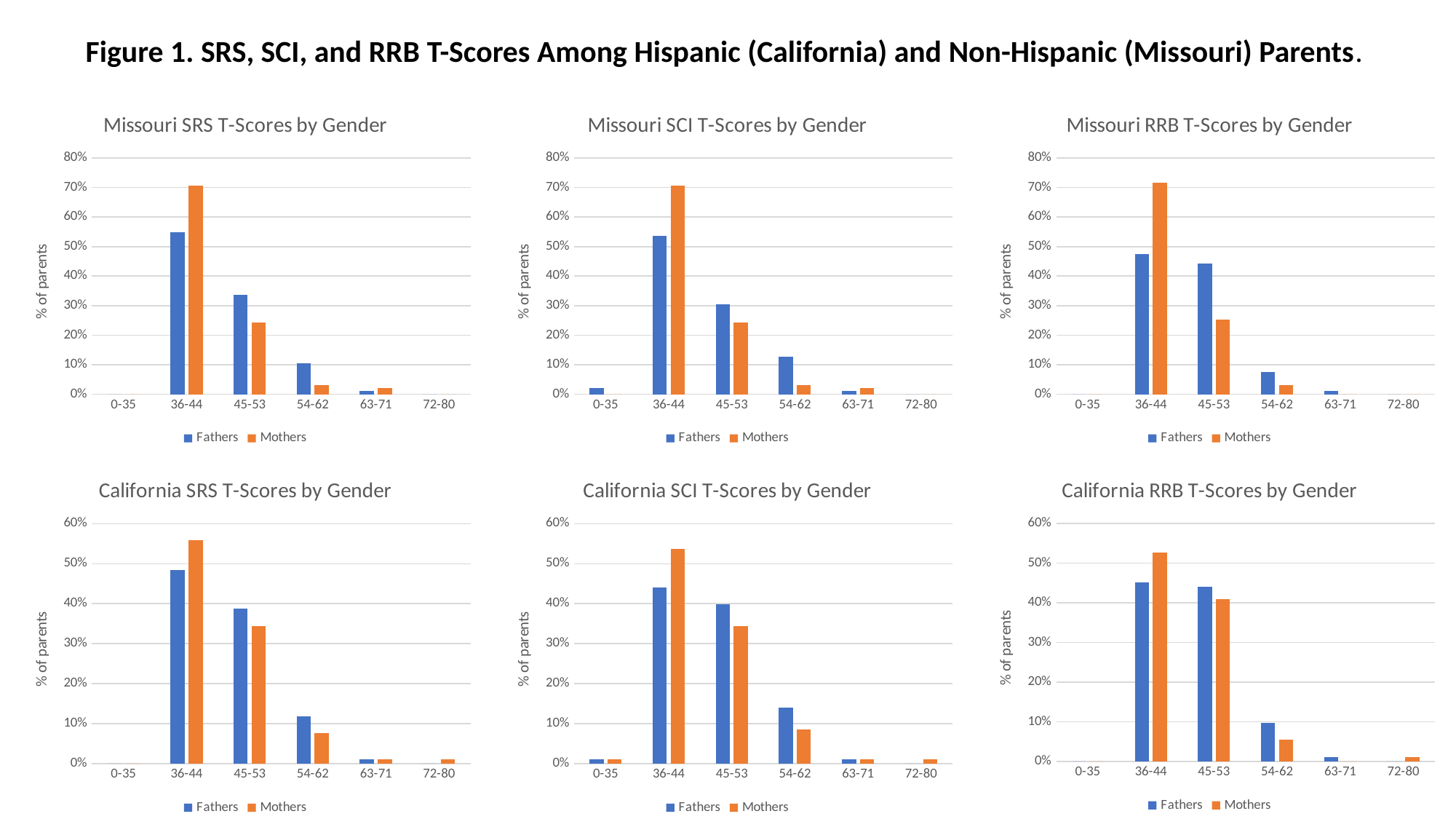

Figure 1. SRS, SCI, and RRB T-Scores Among Hispanic (California) and Non-Hispanic (Missouri) Parents.
### Chart: Missouri SRS T-Scores by Gender
| Category | | |
|---|---|---|
| 0-35 | 0.0 | 0.0 |
| 36-44 | 0.5473684210526316 | 0.7052631578947368 |
| 45-53 | 0.3368421052631579 | 0.24210526315789474 |
| 54-62 | 0.10526315789473684 | 0.031578947368421054 |
| 63-71 | 0.010526315789473684 | 0.021052631578947368 |
| 72-80 | 0.0 | 0.0 |
### Chart: Missouri SCI T-Scores by Gender
| Category | | |
|---|---|---|
| 0-35 | 0.021052631578947368 | 0.0 |
| 36-44 | 0.5368421052631579 | 0.7052631578947368 |
| 45-53 | 0.30526315789473685 | 0.24210526315789474 |
| 54-62 | 0.12631578947368421 | 0.031578947368421054 |
| 63-71 | 0.010526315789473684 | 0.021052631578947368 |
| 72-80 | 0.0 | 0.0 |
### Chart: Missouri RRB T-Scores by Gender
| Category | | |
|---|---|---|
| 0-35 | 0.0 | 0.0 |
| 36-44 | 0.47368421052631576 | 0.7157894736842105 |
| 45-53 | 0.4421052631578947 | 0.25263157894736843 |
| 54-62 | 0.07526881720430108 | 0.031578947368421054 |
| 63-71 | 0.010752688172043012 | 0.0 |
| 72-80 | 0.0 | 0.0 |
### Chart: California SRS T-Scores by Gender
| Category | | |
|---|---|---|
| 0-35 | 0.0 | 0.0 |
| 36-44 | 0.4838709677419355 | 0.5591397849462365 |
| 45-53 | 0.3870967741935484 | 0.34408602150537637 |
| 54-62 | 0.11827956989247312 | 0.07526881720430108 |
| 63-71 | 0.010752688172043012 | 0.010752688172043012 |
| 72-80 | 0.0 | 0.010752688172043012 |
### Chart: California SCI T-Scores by Gender
| Category | | |
|---|---|---|
| 0-35 | 0.010752688172043012 | 0.010752688172043012 |
| 36-44 | 0.44086021505376344 | 0.5376344086021505 |
| 45-53 | 0.3978494623655914 | 0.34408602150537637 |
| 54-62 | 0.13978494623655913 | 0.08602150537634409 |
| 63-71 | 0.010752688172043012 | 0.010752688172043012 |
| 72-80 | 0.0 | 0.010752688172043012 |
### Chart: California RRB T-Scores by Gender
| Category | | |
|---|---|---|
| 0-35 | 0.0 | 0.0 |
| 36-44 | 0.45161290322580644 | 0.5268817204301075 |
| 45-53 | 0.44086021505376344 | 0.40860215053763443 |
| 54-62 | 0.0967741935483871 | 0.053763440860215055 |
| 63-71 | 0.010752688172043012 | 0.0 |
| 72-80 | 0.0 | 0.010752688172043012 |
