# Supplementary material for: Mate selection and current trends in the prevalence of autism
Source: Mol Autism. 2024 Jul 16;15:29. doi: 10.1186/s13229-024-00607-3 (PMC11251233; doi:10.1186/s13229-024-00607-3)
Supplement: Supplementary file 2 — Respective spousal regression lines when dividing the sample into thirds, by autistic trait burden. California (left), Missouri (right). [file 13229_2024_607_MOESM2_ESM.pptx]

## Slide 1
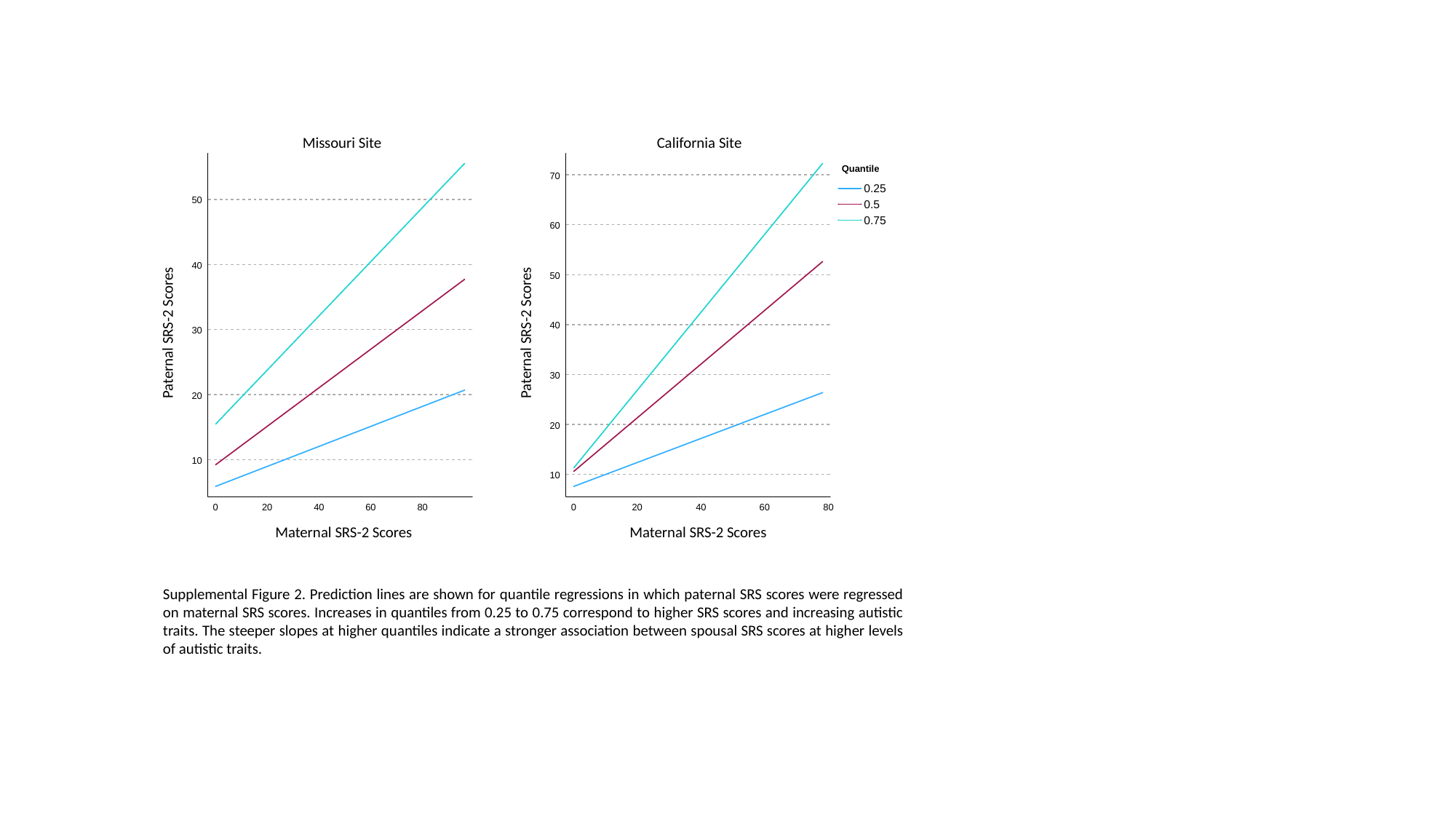

Missouri Site
California Site
Paternal SRS-2 Scores
Paternal SRS-2 Scores
Maternal SRS-2 Scores
Maternal SRS-2 Scores
Supplemental Figure 2. Prediction lines are shown for quantile regressions in which paternal SRS scores were regressed on maternal SRS scores. Increases in quantiles from 0.25 to 0.75 correspond to higher SRS scores and increasing autistic traits. The steeper slopes at higher quantiles indicate a stronger association between spousal SRS scores at higher levels of autistic traits.
