# Supplementary figures and images for: Mate selection and current trends in the prevalence of autism
Source: Mol Autism. 2024 Jul 16;15:29. doi: 10.1186/s13229-024-00607-3 (PMC11251233; doi:10.1186/s13229-024-00607-3)

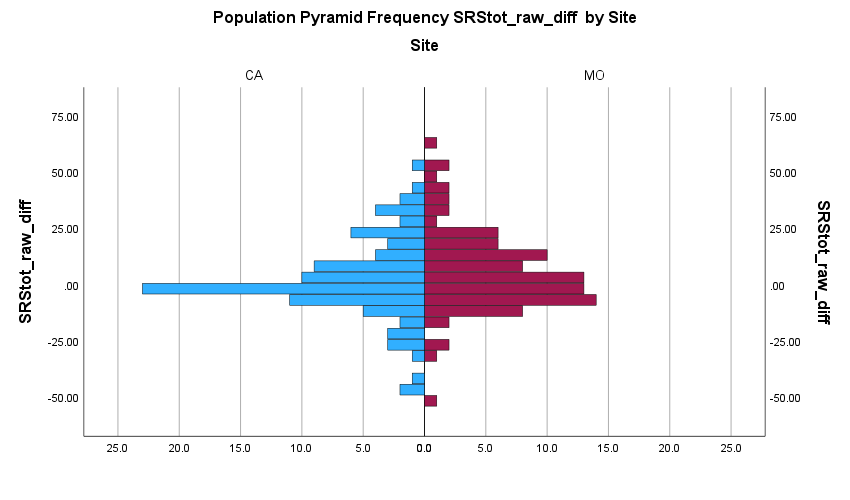
**Supplemental Figure 3. Distributions of Parent Score Differences**


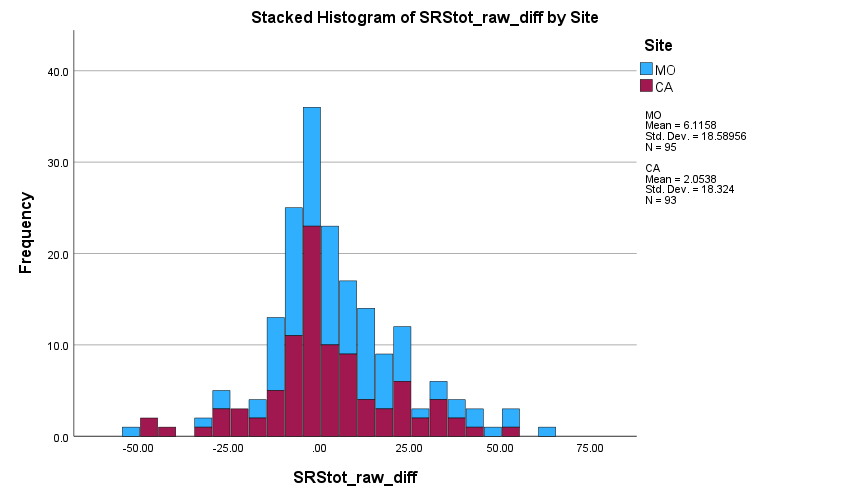

Supplement: Supplementary file 3 — Distributions of Parent Score Differences. [file 13229_2024_607_MOESM3_ESM.docx]
