# Supplementary material for: Mate selection and current trends in the prevalence of autism
Source: Mol Autism. 2024 Jul 16;15:29. doi: 10.1186/s13229-024-00607-3 (PMC11251233; doi:10.1186/s13229-024-00607-3)
Supplement: Supplementary file 7 — Correlations of Parent Scores with Child Scores in Missouri, California, and both sites, respectively (36 or 48 months). [file 13229_2024_607_MOESM7_ESM.docx]

**Supplemental Table 4A. Correlations of Parent Scores with Child Scores in Missouri (36 or 48 mos)**

|  | | SRStot_raw_diff | biparental_mean_raw_SRS | srs2.1: SRS2 Total Raw Score | srs2.2: SRS2 Total Raw Score | srs2_48_or_36.102: SRS2 Total Raw Score | srs2_48_or_36.101: SRS2 Total Raw Score |
| --- | --- | --- | --- | --- | --- | --- | --- |
| SRStot_raw_diff | Pearson Correlation | -- |  |  |  |  |  |
|  | N | 95 |  |  |  |  |  |
| biparental_mean_raw_SRS | Pearson Correlation | .188 | -- |  |  |  |  |
|  | Sig. (2-tailed) | .068 |  |  |  |  |  |
|  | N | 95 | 95 |  |  |  |  |
| srs2.1: SRS2 Total Raw Score | Pearson Correlation | .648^**^ | .870^**^ | -- |  |  |  |
|  | Sig. (2-tailed) | <.001 | <.001 |  |  |  |  |
|  | N | 95 | 95 | 95 |  |  |  |
| srs2.2: SRS2 Total Raw Score | Pearson Correlation | -.424^**^ | .810^**^ | .415^**^ | -- |  |  |
|  | Sig. (2-tailed) | <.001 | <.001 | <.001 |  |  |  |
|  | N | 95 | 95 | 95 | 95 |  |  |
| srs2_48_or_36.102: SRS2 Total Raw Score | Pearson Correlation | -.026 | .289^**^ | .211^*^ | .282^**^ | -- |  |
|  | Sig. (2-tailed) | .801 | .005 | .040 | .006 |  |  |
|  | N | 95 | 95 | 95 | 95 | 95 |  |
| srs2_48_or_36.101: SRS2 Total Raw Score | Pearson Correlation | .046 | .089 | .092 | .054 | .246^*^ | -- |
|  | Sig. (2-tailed) | .659 | .392 | .376 | .600 | .016 |  |
|  | N | 95 | 95 | 95 | 95 | 95 | 95 |
| **. Correlation is significant at the 0.01 level (2-tailed). | | | | | | | |
| *. Correlation is significant at the 0.05 level (2-tailed). | | | | | | | |
| a. Site = MO | | | | | | | |

**Supplemental Table 4B. Correlations of Parent Scores with Child Scores in California (36 or 48 mos)**

|  | | SRStot_raw_diff | biparental_mean_raw_SRS | srs2.1: SRS2 Total Raw Score | srs2.2: SRS2 Total Raw Score | srs2_48_or_36.102: SRS2 Total Raw Score | srs2_48_or_36.101: SRS2 Total Raw Score |
| --- | --- | --- | --- | --- | --- | --- | --- |
| SRStot_raw_diff | Pearson Correlation | -- |  |  |  |  |  |
|  | N | 93 |  |  |  |  |  |
| biparental_mean_raw_SRS | Pearson Correlation | -.084 | -- |  |  |  |  |
|  | Sig. (2-tailed) | .421 |  |  |  |  |  |
|  | N | 93 | 93 |  |  |  |  |
| srs2.1: SRS2 Total Raw Score | Pearson Correlation | .460^**^ | .846^**^ | -- |  |  |  |
|  | Sig. (2-tailed) | <.001 | <.001 |  |  |  |  |
|  | N | 93 | 93 | 93 |  |  |  |
| srs2.2: SRS2 Total Raw Score | Pearson Correlation | -.567^**^ | .869^**^ | .470^**^ | -- |  |  |
|  | Sig. (2-tailed) | <.001 | <.001 | <.001 |  |  |  |
|  | N | 93 | 93 | 93 | 93 |  |  |
| srs2_48_or_36.102: SRS2 Total Raw Score | Pearson Correlation | -.082 | .237^*^ | .167 | .236^*^ | -- |  |
|  | Sig. (2-tailed) | .434 | .022 | .110 | .023 |  |  |
|  | N | 93 | 93 | 93 | 93 | 93 |  |
| srs2_48_or_36.101: SRS2 Total Raw Score | Pearson Correlation | -.090 | .217^*^ | .145 | .224^*^ | .468^**^ | -- |
|  | Sig. (2-tailed) | .390 | .037 | .165 | .031 | <.001 |  |
|  | N | 93 | 93 | 93 | 93 | 93 | 93 |
| **. Correlation is significant at the 0.01 level (2-tailed). | | | | | | | |
| *. Correlation is significant at the 0.05 level (2-tailed). | | | | | | | |
| a. Site = CA | | | | | | | |

**Supplemental Table 4C. Correlations of Parent Scores with Child Scores for Both Sites (36 or 48 mos)**

|  | | SRStot_raw_diff | biparental_mean_raw_SRS | srs2.1: SRS2 Total Raw Score | srs2.2: SRS2 Total Raw Score | srs2_48_or_36.102: SRS2 Total Raw Score | srs2_48_or_36.101: SRS2 Total Raw Score |
| --- | --- | --- | --- | --- | --- | --- | --- |
| SRStot_raw_diff | Pearson Correlation | -- |  |  |  |  |  |
|  | N | 188 |  |  |  |  |  |
| biparental_mean_raw_SRS | Pearson Correlation | .031 | -- |  |  |  |  |
|  | Sig. (2-tailed) | .676 |  |  |  |  |  |
|  | N | 188 | 188 |  |  |  |  |
| srs2.1: SRS2 Total Raw Score | Pearson Correlation | .545^**^ | .855^**^ | -- |  |  |  |
|  | Sig. (2-tailed) | <.001 | <.001 |  |  |  |  |
|  | N | 188 | 188 | 188 |  |  |  |
| srs2.2: SRS2 Total Raw Score | Pearson Correlation | -.507^**^ | .846^**^ | .446^**^ | -- |  |  |
|  | Sig. (2-tailed) | <.001 | <.001 | <.001 |  |  |  |
|  | N | 188 | 188 | 188 | 188 |  |  |
| srs2_48_or_36.102: SRS2 Total Raw Score | Pearson Correlation | -.085 | .274^**^ | .186^*^ | .282^**^ | -- |  |
|  | Sig. (2-tailed) | .246 | <.001 | .011 | <.001 |  |  |
|  | N | 188 | 188 | 188 | 188 | 188 |  |
| srs2_48_or_36.101: SRS2 Total Raw Score | Pearson Correlation | -.019 | .153^*^ | .118 | .142 | .347^**^ | -- |
|  | Sig. (2-tailed) | .793 | .036 | .106 | .052 | <.001 |  |
|  | N | 188 | 188 | 188 | 188 | 188 | 188 |
| **. Correlation is significant at the 0.01 level (2-tailed). | | | | | | | |
| *. Correlation is significant at the 0.05 level (2-tailed). | | | | | | | |

**Note:** Parent-child score associations are consistent with influence of familial factors on child scores and prior literature – parent score exhibits a stronger association with the child’s score than does the difference between parent scores.
